# Low Acyl Gellan as an Excipient to Improve the Sprayability and Mucoadhesion of Iota Carrageenan in a Nasal Spray to Prevent Infection With SARS-CoV-2

**DOI:** 10.3389/fmedt.2021.687681

**Published:** 2021-06-16

**Authors:** Thomas E. Robinson, Richard J. A. Moakes, Liam M. Grover

**Affiliations:** Healthcare Technologies Institute, School of Chemical Engineering, University of Birmingham, Birmingham, United Kingdom

**Keywords:** antiviral polymer, nasal spray, mucoadhesion, gellan, carrageenan, COVID-19

## Abstract

The COVID-19 global pandemic, as well as the widespread persistence of influenza and the common cold, create the need for new medical devices such as nasal sprays to prevent viral infection and transmission. Carrageenan, a sulfated polysaccharide, has a broad, non-pharmacological antiviral capacity, however it performs poorly in two key areas; spray coverage and mucoadhesion. Therefore gellan, another polysaccharide, was investigated as an excipient to improve these properties. It was found that viscoelastic relaxation time was the key predictor of spray coverage, and by reducing this value from 2.5 to 0.25 s, a mix of gellan and carrageenan gave more than four times the coverage of carrageenan alone (*p* < 0.0001). Gellan also demonstrated enhanced adhesion to a mucus analog that increased significantly with time (*p* < 0.0001), suggesting the development of specific gellan–mucin interactions. This property was conferred to carrageenan on mixing the two polymers. Together, this data suggests that gellan is a promising excipient to improve both sprayability and mucoadhesion of carrageenan for use in antiviral nasal sprays.

## Introduction

The spread of severe acute respiratory syndrome coronavirus 2 (SARS-CoV-2) has caused over a quarter of a billion cases of COVID-19 worldwide, leading to more than 2.7 million deaths, as of 26^th^ March 2021 (1). While transmission of viruses is complex, with multiple potential pathways including direct contact (person-to-person transmission) and indirect contact (transmission through contaminated objects), airborne transmission is thought to be the predominant route. Indeed, although a new strain, it seems to follow a similar, airborne, mechanism of transmission as the SARS-CoV-1 virus in the early 2000s (2), as well as other respiratory viruses (3). Although the exact routes of transmission for SARS-CoV-2 are still contentious, analysis of known “superspreading events” within social situations, including a restaurant and a choir practice, strongly suggest that airborne transmission plays a significant role (4–7). Droplets containing infective viral loads can travel up to 250 m before hitting the ground, depending on droplet size, velocity, and air flow, with spreading in poorly ventilated indoor areas thought to be the most prevalent (8, 9).

**Figure 1:**
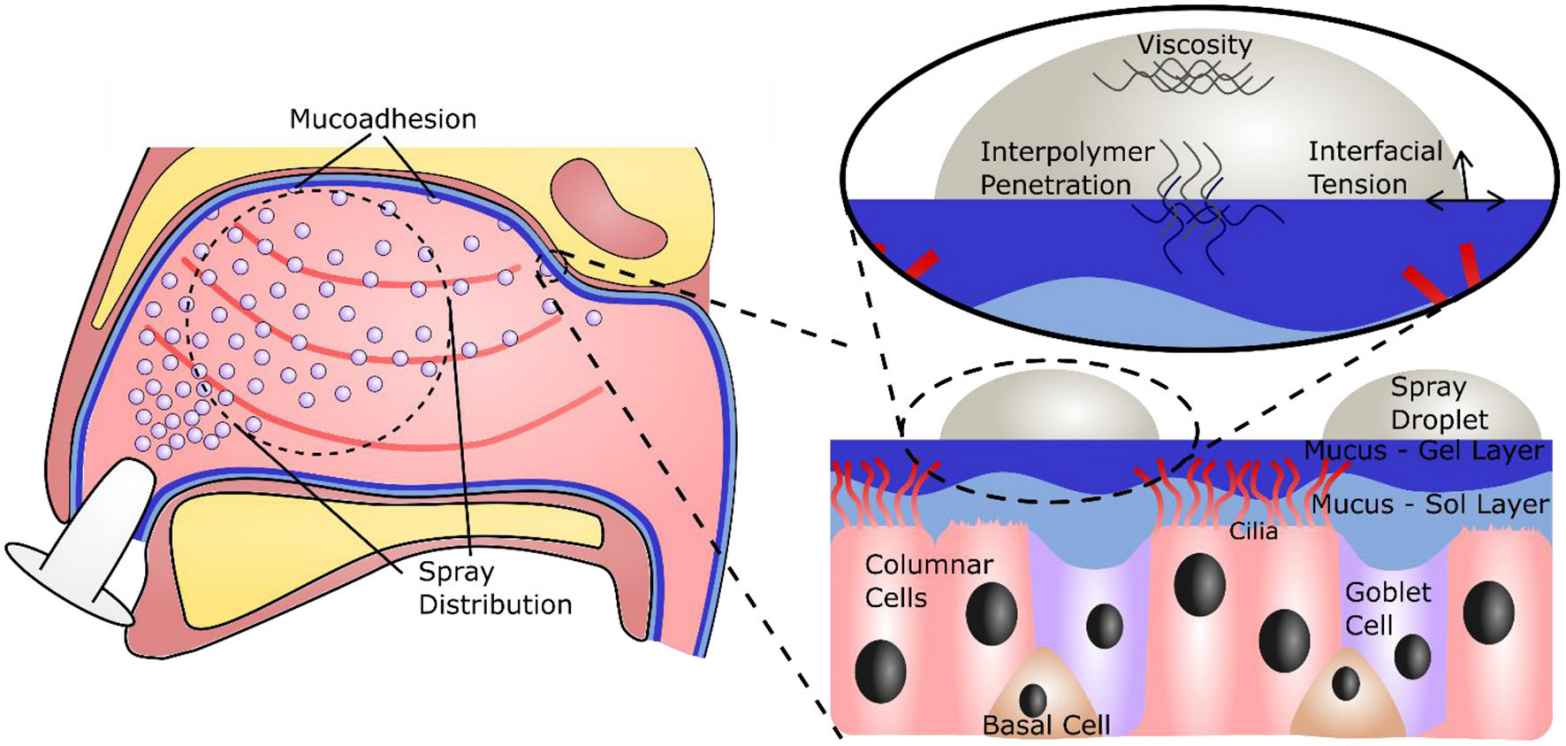
Illustration of the nasal cavity, showing the necessity for a large, even spray distribution and subsequent adhesion to the nasal mucus. A focus on the nasal mucosa shows the specialized epithelial cells and the mucus bi-layer they produce. A further focus shows the important forces on and within a droplet that will govern retention time on the mucosa.

Air is primarily inhaled through the nose, on average 10,000 L per day (10, 11). The nasal passage is specialized to condition the temperature and humidity of the air, as well as remove contaminants such as particles and droplets (12, 13). The specialized cells in the nasal epithelium that perform these functions include columnar cells, both ciliated and non-ciliated, goblet cells, and basal cells, and one of the key mechanisms for this is the production and transport of mucus (12, 14) (Figure 1). Nasal mucus is a viscoelastic material that covers the epithelium, conditioning the air, and acting as a physical barrier against particles, microbes, and viruses (15). It consists of two phases; a less viscous sol layer beneath a more viscous gel layer. Mainly composed of water, the viscoelastic properties of the mucus are mainly attributed to mucins, high molecular weight glycoproteins that make up around 2 wt% of the material (15–17). In recent years the advantages of delivering actives through mucosal tissues have been recognized, leading to the development of and use of excipients that will adhere to the mucus layer for extended periods (18). Several properties determine retention time on the mucosa, including droplet size, viscosity, and interfacial tension. An additional factor is mucoadhesion, the interpenetration and attractive interaction between polymers in the formulation and those in the mucus (13, 19).

**Figure 2:**
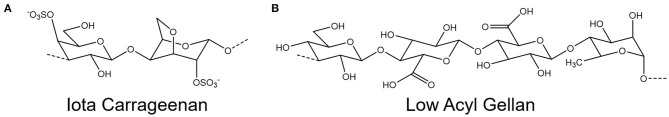
Chemical structure of the repeating units of **(A)** iota carrageenan and **(B)** low acyl gellan.

The nasal cavity has a volume of between 15 and 19 ml, and a macroscopic surface area of 150–180 cm^2^, however the presence of microstructures such as microvilli on the columnar cells drastically increase this surface area to around 96,000 cm^2^ (13, 20, 21). While this provides a large area for filtration, it also presents a large target for viral infiltration; evidenced through reported uptake of SARS-Cov-2 within the nasal epithelium (22). Although emerging technologies are being seen to help protect this area (23–25), adequate devices to prevent contraction of airborne viruses and stop further spread are still needed. One option for such a device is a spray formulation, applied directly to the nasal cavity, to bolster the natural antiviral function of the mucosa (26). Such a formulation, in addition to exhibiting a potent ability to prevent viral infection and transmission, should give a large, even spray plume, to fully coat the surface of the nasal cavity, and adhere to the mucus layer, so that it can be retained and extend antiviral protection (Figure 1). A promising candidate for new antiviral formulations is iota carrageenan (Figure 2A), a sulfated polysaccharide derived from seaweeds of the *Rhodophycea* family. Carrageenans have been widely applied within the food industry as thickeners and stabilizing agents, but have more recently found their way into the pharmaceutical and cosmetic industry (27). Further, carrageenans have been shown to possess a broad, non-specific antiviral capacity, able to inhibit viruses including the common cold (28–30), influenza (31, 32), and SARS-CoV-2 (32–34). However, at clinically relevant viscosities, carrageenan demonstrates poor sprayability (26). An excipient is therefore required to improve the material properties of carrageenan for use as an antiviral spray device.

In this paper, we investigate gellan (Figure 2B), another food and pharmaceutical grade polysaccharide, as an excipient to improve the relatively poor performance of the antiviral carrageenan in two key areas: sprayability and mucoadhesion. Systematic control over the formulation has been used to understand the mechanisms that facilitate enhanced spray coverage, and relate this to relevant material properties, namely shear viscosity, surface tension, and viscoelasticity. A similar approach has been used to understand adhesion, and the physical properties which drive this adhesion have been investigated. The specific contribution of mucoadhesion is then analyzed, by comparing adhesion to a surface without mucins present, and hypothesizing that mucoadhesion, the interpenetration and interaction of formulation and mucin polymers, will develop over a relatively long time period, compared to viscous and interfacial forces. This hypothesis is then explored by a rheological measurement of the development of the mucoadhesive force over time, and measurement of contact angle over time.

## Materials and Methods

### Materials

Gellan gum (low acyl) (Kelcogel CGLA) was purchased from CPKelco; deionized water (Type 1, Millipore); Iota carrageenan, porcine gelatin (Type A), porcine stomach mucins (Type II) were all purchased from Sigma Aldrich; black dye (Quink, Parker).

### Preparation of Spray Solutions

Gellan dispersions were prepared by dispersing 0.5 and 1% (w/v) in deionized water and agitating until fully dispersed using a magnetic stirrer, under ambient conditions (20°C). Iota carrageenan solutions were prepared in a similar manner, at 0.5% (w/v). A composite blend was obtained by first preparing the individual gellan and carrageenan [both 0.5% (w/v)] dispersions and subsequently mixing at a ratio of 1:1, resulting in a final concentration of 0.25% (w/v) gellan and 0.25% (w/v) carrageenan. Dispersions were then left overnight to fully hydrate at room temperature prior to use.

### Spraying

Spray coverage was assessed by staining the spray formulations with black dye [1% (v/v)] and mechanically spraying them onto paper. Formulations were loaded into standard commercial handheld spray bottles, and primed by depressing the actuator three times. The formulation was then sprayed horizontally, at a distance of 15 cm, at a piece of suspended A4 paper. The residue was allowed to dry, before being scanned at 600 dpi. Images were then loaded into ImageJ, cropped (4,000 by 4,000 px square), and a threshold applied. Particle analysis was then carried out to calculate the percentage coverage of the cropped square, and this was converted back to a real surface area.

### Preparation of Gelatin/Mucin Substrates

Gelatin [10 and 20% (w/v)] solutions were prepared by first dissolving in deionized water at 80°C. A mucin [10% (w/v)] solution was prepared at ambient temperature through the addition of powder to deionized water under constant agitation for 24 h. Gelatin only substrates were produced using a casting method; 80°C gelatin [5 ml, 10% (w/v)] solution was pipetted into a plastic petri dish and allowed to cool for 24 h prior to use. Gelatin–mucin substrates were prepared by first mixing gelatin [20% (w/v)] (whilst still hot) with the mucin solution at a ratio of 1:1, giving a final concentration of 10% (w/v) gelatin and 5% (w/v) mucin. Casts were produced in the same manner as the gelatin only, and kept for 24 h prior to testing.

### Rheology

All rheological characterization was carried out on a rotational rheometer (Kinexus Ultra, Netzsch GmbH), at 25°C. Shear viscosity profiles were obtained using a cone and plate geometry (diameter 40 mm, angle 4°) in rate-controlled mode between a shear rate of 0.01 and 1,000 s^−1^, over 5 min. Datasets were fit with a Sisko model (35):


$$\begin{array}{l} \mu =\mu_{\infty }+K{\overset{∙}{\gamma }}^{n-1} \end{array}$$

Where μ is the viscosity (Pa.s), μ_∞_ is the viscosity at infinite shear (Pa.s), $\overset{∙}{\gamma }$ is the shear rate (s^−1^), *n* is the rate index (–), and *K* is the consistency index (Pa.s^n^).

Small deformation rheology was conducted using a parallel plate geometry (diameter 50 mm, gap 0.5 mm). A strain (0.5%) common to all formulations' linear viscoelastic regions was obtained by first preforming amplitude sweeps. Frequency sweeps were then performed, at 0.5% strain, between 0.1 and 10 Hz, and the crossover point (G′ = G′′) was determined.

To study mucoadhesion, a gel surface (gelatin or mucin gelatin) was used as the bottom plate, coupled with a cone (diameter 40 mm, angle 4°). After loading, the formulation was rejuvenated at 10 s^−1^ for 10 s to ensure homogeneity between loading. A single frequency oscillatory test, at 1 Hz and 0.5% strain, was used to measure the change in the material structure over time.

### Surface Tensions and Wettability

Surface tension of the formulations in air was determined by dispensing the formulation (7 μl) from a pipette to the point just prior to detachment, to form a hanging drop, and imaging (USB microscope, Veho). Images were analyzed using an image analysis package (ImageJ), by first selecting a region of interest around the drop and using the pendant drop plugin, as described by Daerr and Mogne (36).

Wettability was measured through assessment of the contact angle between the air-substrate interface. Measurements were obtained by pipetting 7 μl of the formulation onto either a gelatin or gelatin–mucin substrate. Images were obtained at three time points (*t* = 0, *t* = 120, and *t* = 300 s) using a USB microscope. Images were analyzed using an image analysis package (ImageJ), by first selecting a region of interest around the drop and using DropSnake method to quantify the left and right contact angles as describe by Stalder et al. (37, 38). An average of the two angles was then used to describe the overall contact angle for the drop.

### Mucoadhesion on an Inclined Surface

Adhesion of the formulation to an inclined surface was performed by pipetting 100 μl of the formulation onto either a gelatin or gelatin–mucin plate. Three samples were applied to the same substrate at 120 and 300 s intervals (covering the samples between additions). Once the third sample was applied the substrate was immediately placed on a 45° surface, the time taken for the droplets to flow 20 mm down the surface was recorded.

### Statistical Analysis

All data is reported as mean ± standard deviation (*SD*) with *n* = 3. Data sets were analyzed using either a one- or two-way analysis of variance test (ANOVA) as appropriate, and applying a *post-hoc* Tukey's multiple comparisons test to compare groups when required. In all cases, *p* > 0.05 was defined as not significant (ns), and the following notation was used for significant results: *p < 0.05, **p < 0.01, ***p < 0.001, and ****p < 0.0001.

## Results

### Spray Dispersion

**Figure 3:**
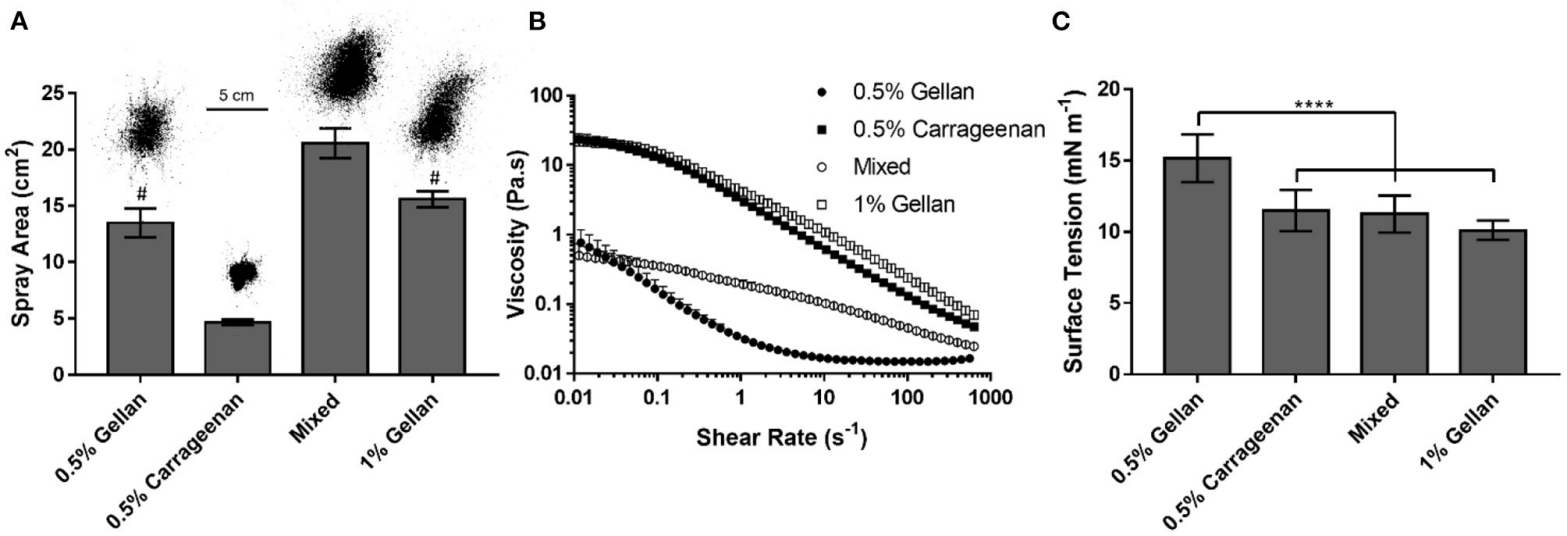
**(A)** Spray area covered by each formulation, with a representative image of each distribution. The mixed system contains 0.25% gellan and 0.25% carrageenan. **(B)** Shear rate ramps of each formulation. **(C)** Surface tension of each formulation. All graphs show mean ± *SD* (*n* = 3). Panels **(A,C)** show the results of ordinary one-way ANOVAs with a *post-hoc* Tukey's multiple comparisons test. In **(A)**, all columns differ significantly from each other except 0.5% gellan and 1% gellan, indicated by #.

The ability of the formulations to adequately cover the nasal mucosa was examined by analyzing their spray distribution and surface coverage (Figure 3A). The spray pattern for all the formulations displayed a central zone with continuous coverage, surrounded by satellite droplets. However, the size of this central zone and the extent of satellite droplets was dependent on spray material, with gellan formulations giving larger profiles compared to carrageenan only. Quantification of the surface coverage highlighted that at the same polymer concentration [0.5% (w/v)], the gellan formulation gave 2.9 times the coverage of carrageenan (*p* < 0.0001). Interestingly, the mixture of the polymers, containing 0.25% (w/v) gellan and 0.25% (w/v) carrageenan, displayed an even greater coverage than either single polymer system (*p* < 0.0001), and was 4.4-fold the coverage of 0.5% (w/v) carrageenan. The effect of formulation viscosity on sprayability was studied through shear rheology (Figure 3B). All formulations displayed shear thinning behavior, and application of the Sisko model showed that 0.5% (w/v) gellan had a consistency index, *K*, of 0.02 ± 0.01, carrageenan had a *K* of 3.15 ± 0.24, and the mixed system had an intermediate *K*-value of 0.22 ± 0.03. To negate the effect of viscosity on sprayability, a second gellan solution [1% (w/v)] was prepared, with a similar shear viscosity profile as the carrageenan (a *K*-value of 4.29 ± 0.26). The spray coverage of the isoviscous gellan was still significantly higher than the carrageenan (*p* < 0.0001), being similar to the 0.5% (w/v) gellan (*p* > 0.05). The effect of surface tension on formulation sprayability was also studied (Figure 3C). 0.5% (w/v) gellan displayed a surface tension of 15.2 ± 1.7 mN m^−1^, significantly higher than both the carrageenan and mixture (*p* < 0.0001), which had surface tensions of 11.5 ± 1.4 and 11.3 ± 1.3 mN m^−1^, respectively. Increasing the gellan concentration to 1% (w/v) lowered the surface tension to 10.1 ±0.7 mN m^−1^, the lowest of all the samples.

**Figure 4:**
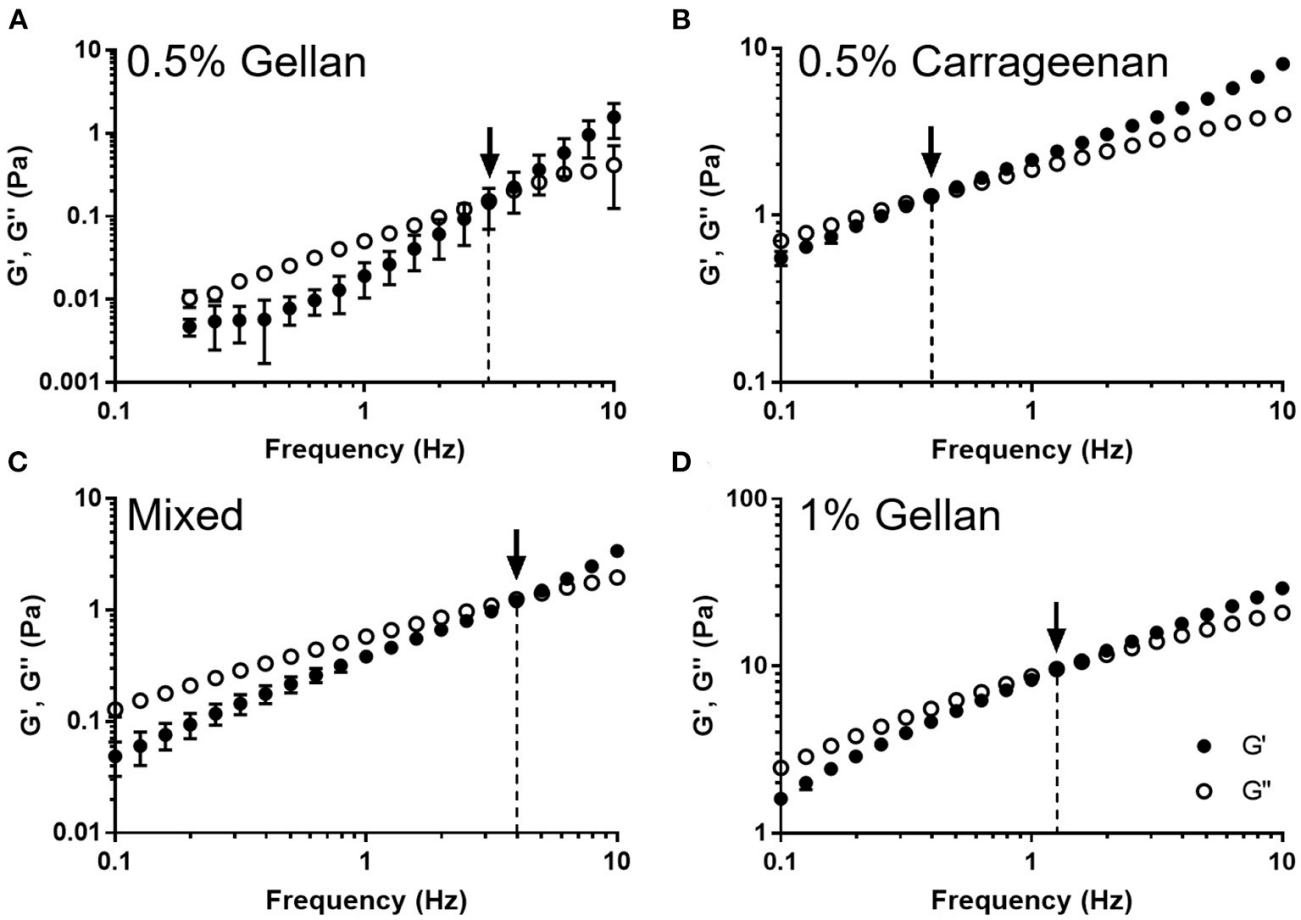
The storage modulus (G′) and loss modulus (G′′) as a function of frequency for **(A)** 0.5% gellan, **(B)** 0.5% carrageenan, **(C)** the mixed system, and **(D)** 1% gellan. Arrows indicate the point at which G′ = G′′, and the dashed lines show the frequency at which this occurs. Graphs show mean ± *SD* (*n* = 3).

The viscoelastic properties of the formulations were studied through dynamic oscillatory tests (Figure 4). All formulations displayed frequency dependent behavior indicative of viscoelastic liquids; being viscously dominated (G′ < G′′) at low frequency, undergoing a crossover (G′ = G′′), and becoming elastically dominated (G′ > G′′) at higher frequencies. The crossover frequency was dependent on the formulation, with carrageenan transitioning at the lowest frequency, 0.4 Hz, gellan transitioning between 1 and 3 Hz, and the mixture transitioning at 4 Hz, a ten-fold higher frequency than the carrageenan.

### Mucoadhesion

**Figure 5:**
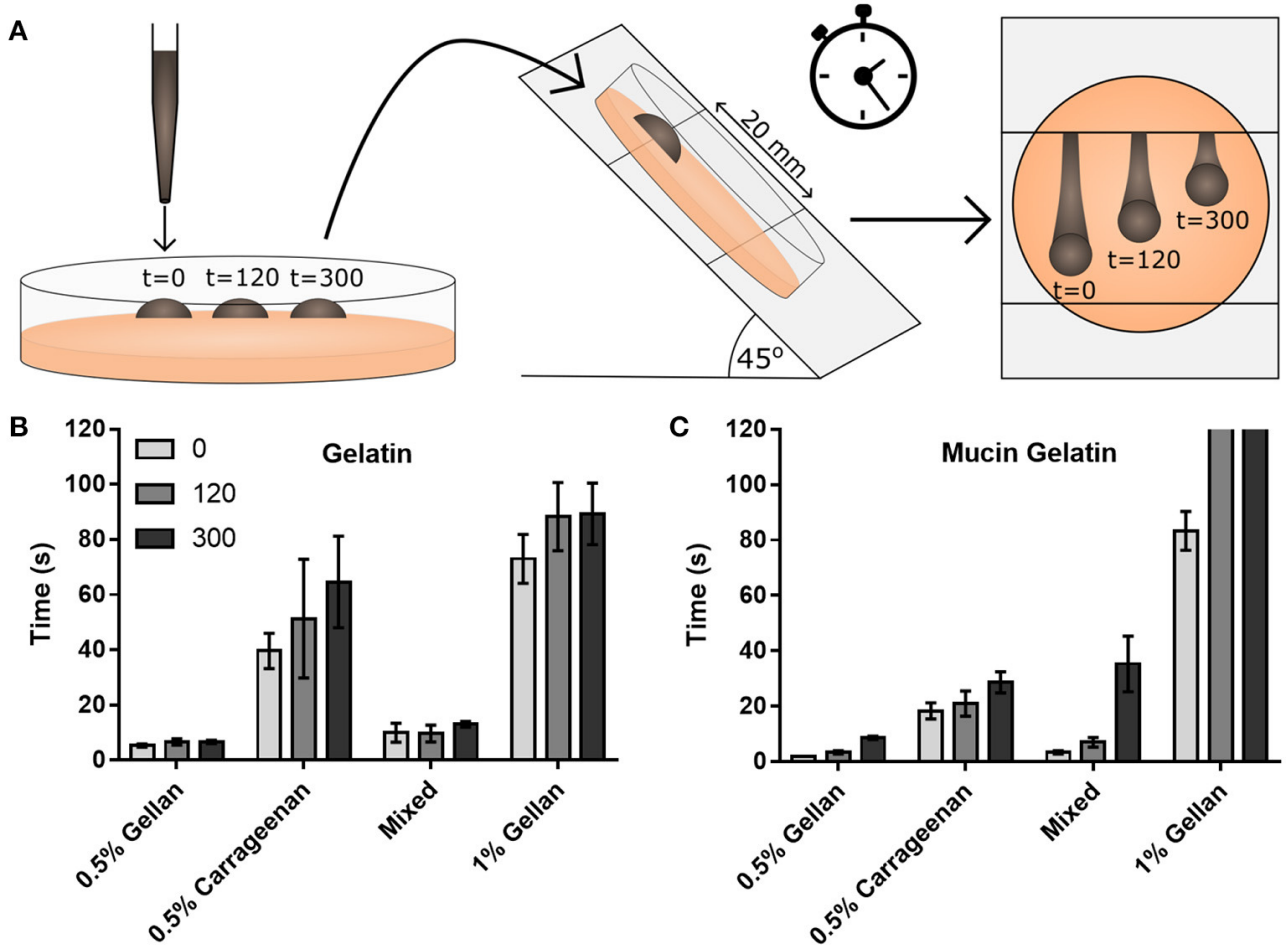
**(A)** A schematic of the drop flow adhesion study, where drops of formulation are pipetted onto a gelatin only or mucin gelatin surface, allowed to adhere for 0, 120, or 300 s, before being tilted at 45°, and their time to travel 20 mm measured. Flow times are displayed for **(B)** the gelatin only substrate and **(C)** the mucin functionalized substrate. Note that in **(C)**, drops of 1% gellan did not flow at all on tilting at 120 and 300 s. **(B,C)** show mean ± *SD* (*n* = 3).

Adhesion between the mucus and the spray was studied using gelatin substrates, either functionalized with mucins (mucin gelatin) or without (gelatin only). Time dependent interactions were probed by allowing the formulation to rest on the substrate for 300, 120, or 0 s prior to inclining at 45° and measuring the time taken for the droplet to flow 20 mm (Figure 5A). 0.5% (w/v) gellan took the shortest time to flow down the gelatin only substrate, followed by the mixture, then carrageenan, and the 1% (w/v) gellan (Figure 5B). On the combined mucin gelatin surfaces, all formulations other than the 1% (w/v) gellan showed a decrease in running time at *t* = 0 (Figure 5C). There also appeared to be a more pronounced increase in running time with application time for formulations that contained gellan. This was most obvious for the 1% (w/v) gellan, where drops that had been applied for 120 s or more did not flow at all on tilting.

**Figure 6:**
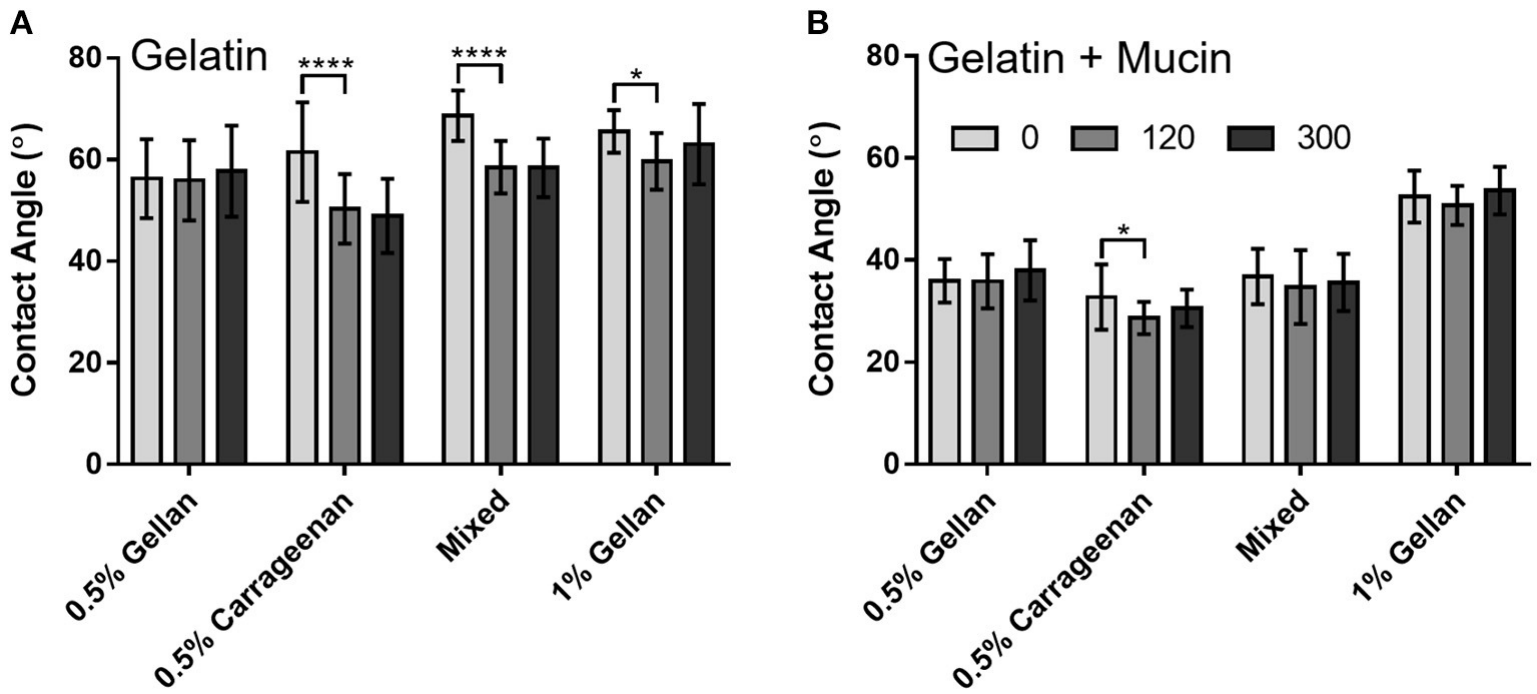
Contact angle measurements of each formulation over time, on **(A)** gelatin only and **(B)** mucin gelatin plates. Graphs show mean ± *SD* (*n* = 3), and show the results of a two-way ANOVA with a *post-hoc* Tukey's multiple comparisons test.

Interfacial forces between the formulation and the surfaces were studied through contact angle. In all cases, the contact angle was higher on the gelatin only surface (Figure 6A) than on the mucin functionalized surface (Figure 6B). Gellan systems, despite showing a time-dependent relationship for flowing, did not show the same trend for wettability, with no significant difference in contact angle at 300 s, irrespective of substrate. This was not the case for formulations containing carrageenan on the gelatin substrate, with the contact angle of carrageenan only and the mixed system decreasing by 11.1° and 10.1°, respectively, at 120 s (*p* < 0.0001). These formulations appear to equilibrate within this time, as no change is seen thereafter. While the presence of mucin clearly decreases the contact angle for all formulations, this angle did not change significantly at 300 s.

**Figure 7:**
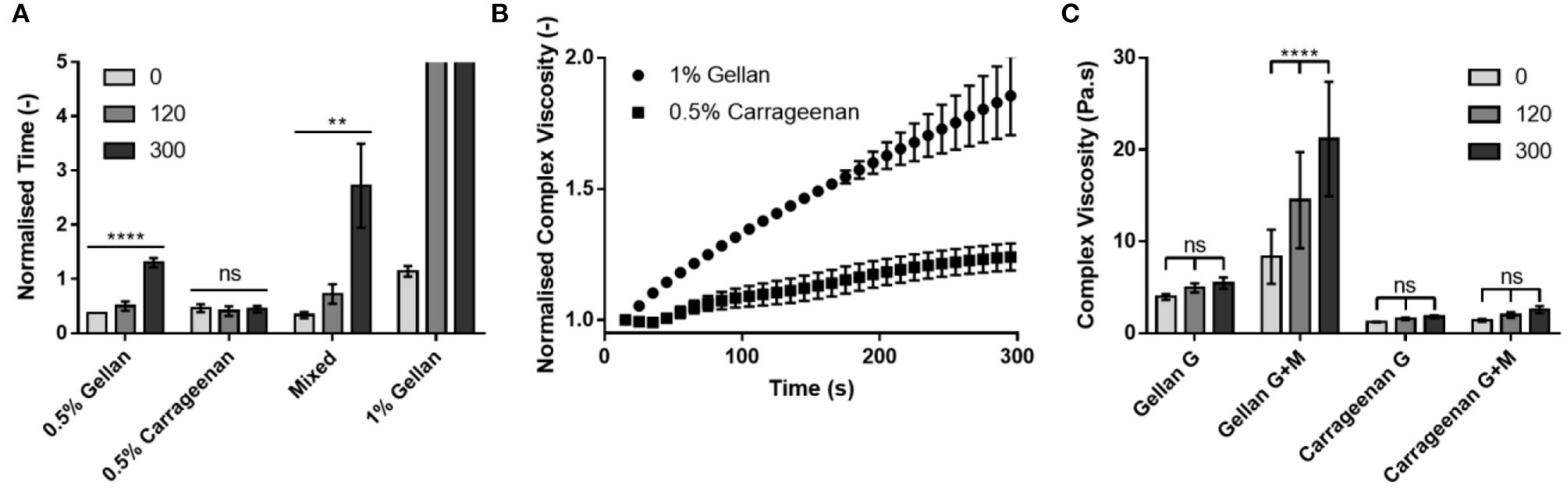
**(A)** The flow time of each formulation on the mucin-gelatin surface, normalized by that on the gelatin only surface at each application time. **(B)** The complex viscosity of 1% gellan and 0.5% carrageenan on a mucin gelatin surface over time, normalized for initial complex viscosity, and complex viscosity on a gelatin only surface at each time point. **(C)** Complex viscosity of each formulation on gelatin (G) and mucin gelatin (G+M), at 0, 120, and 300 s. Note that in **(A)**, drops of 1% gellan did not flow at all on tilting at 120 and 300 s. Graphs show mean ± *SD* (*n* = 3). Panel **(A)** shows the results of ordinary one-way ANOVAs performed on each formulation. Panel **(C)** shows the results of a two-way ANOVA with a *post-hoc* Tukey's multiple comparisons test.

To deconvolute the role of the mucin functionalization of the substrate on adhesion of the formulations, the contribution of viscosity and formulation–gelatin interactions were removed by normalizing the flow time on the mucin gelatin (Figure 5C) by that of the gelatin only (Figure 5B) at each time point (Figure 7A). This highlighted the dependence of running time on application time for the 0.5% (w/v) gellan system, as the relative time taken to flow down the mucin gelatin substrate increased significantly (*p* < 0.0001), by a factor of 3.5 over 300 s. A similar trend was observed for the mixed system, where the running time increased more than eight-fold over 300 s (*p* < 0.01), and for 1% (w/v) gellan, where the drops did not flow at all on the gelatin mucin surface at 120 and 300 s, which may be interpreted as in infinite increase. Conversely, 0.5% (w/v) carrageenan showed no time dependency, with no significant differences in flow time with application time (*p* > 0.05).

To better understand the time dependent nature of the formulations, small deformation rheology was used to probe the changes in mechanical properties arising from interactions between the formulations and the substrates. Formulations were placed between a plate and gelatin-based substrate, rejuvenated via shear, and the change in complex viscosity studied over 300 s. The changes in complex viscosity on the mucin gelatin substrate, relative to those on gelatin only, were plotted in order to understand the development of interactions between gellan or carrageenan and mucin (Figure 7B). Both 1% (w/v) gellan and 0.5% (w/v) carrageenan showed a linear (*R*^2^ = 0.99 and 0.98, respectively) increase in relative complex viscosity as a function of time. However, gellan displayed a gradient more than three-fold that of carrageenan, resulting in a relative increase of 85% for gellan, and only 24% for carrageenan, over 300 s. Indeed, comparison of the non-normalized values showed that gellan had a greater complex viscosity than carrageenan at all-time points and on both substrates (Figure 7C). However, only gellan on the mucin gelatin substrate displayed a significant increase in complex viscosity over time (*p* < 0.0001).

## Discussion

The widespread persistence of respiratory viruses (influenza, the common cold, etc.) highlights a general lack of effective interventions, a reality made more pronounced with the emergence of the COVID-19 pandemic. New devices to prevent viral infection, as well as subsequent transmission, by directly targeting the nasal passage and providing an antiviral capacity, would provide an additional line of defense to the current protective arsenal: face masks, hand sanitization, and social distancing. In response, numerous nasal sprays have been developed seeking translation into over-the-counter products to reduce viral loads, many of which have utilized iota carrageenan (28, 29, 39–41). In addition to its potent antiviral properties, carrageenan is advantageous because it already holds regulatory approval, and is not absorbed through the mucosa or metabolized (30), speeding up translation through the regulatory pathways. However, at polymer concentrations which support clinically relevant viscosities, carrageenan-based sprays have a tendency to “jet,” providing poor coverage of the nasal mucosa (26); an observation also made in this study.

In order to improve the sprayability of carrageenan, gellan, another natural polysaccharide which holds similar regulatory approval and translational advantages to carrageenan (42–44), was investigated as an excipient. Gellan demonstrated a near three-fold increase in spray coverage over carrageenan, at the same polymer concentration (0.5% (w/v), and combining the two polymers gave an even greater distribution. Important properties of the polymer formulations were mechanistically probed, to understand which affected sprayability. Viscosity, through the dimensionless Reynold's number, is often used in correlations to predict “spray angle,” a parameter which directly affects coverage (45–47). However, a 1% (w/v) gellan dispersion, which displayed the same viscosity profile as carrageenan, still sprayed significantly better, suggesting viscosity is not a good predictor of spray coverage for these systems. Surface tension, which plays a key role in film destabilization and droplet formation during spraying (48, 49), also showed no obvious correlation with spray coverage, as the 1% (w/v) gellan, 0.5% (w/v) carrageenan, and mixed system, despite having similar surface tension values, all displayed significantly different spray coverage values to each other.

The lack of dependence on viscosity and surface tension suggested that spray distribution was not related to the ability of the polymers to structure the water or stabilize the air–water interface, respectively, but may be due to interactions between the polymers themselves. Sprayability was thus studied as a function of the dynamic viscoelastic behavior of the formulations. All systems behaved as viscoelastic liquids, transitioning from being viscously dominated at low frequencies (G′ < G′′) to elastically dominated at higher frequencies (G′ > G′′) (50, 51). This is typical of associative polymer networks, where reversible polymer–polymer interactions are formed (52, 53). The frequency at the crossover point (G′ = G′′) is the inverse of the relaxation time of the system. It is suggested that sprayability is a direct consequence of relaxation time as systems with a longer relaxation time, such as carrageenan, will act “solid like” during spraying, hindering disruption, and leading to a narrower distribution. This explanation accounts for the trend of increased spray distribution from carrageenan, to the gellan systems, to the mixture, whose relaxation times were 2.5 s > 0.79–0.32 s > 0.25 s, respectively. The interesting properties of the mixed system may arise because the gellan and carrageenan mixture forms a phase separated blend (54, 55). The intermediate viscosity of the mixture may arise because the two polymers structure water separately, rather than forming synergistic effects. However, if the two components are forming discrete fluid elements, this may reduce long range ordering, and by not interacting with each other will dilute the number of inter-polymer interactions. Both of these effects may lead to a faster relaxation time, and thus greater sprayability, as there are fewer interactions to be broken, and interactions within the discrete elements may not need to be broken if they are smaller than the formed droplets.

Following spraying into the nasal cavity, it is important that the formulations adhere to the mucosa, to give prolonged antiviral activity. Several forces will impact adhesion and retention time, including viscosity, interfacial tension, and mucoadhesion. Viscosity of entangled polyelectrolyte solutions can be modified relatively simply by changing properties such as polymer concentration, ion concentration, and pH (56–59). Interfacial tension is more difficult to control, however the formulations studied all showed low contact angles on mucin functionalized surfaces, indicative of good wettability, and spreading on the mucosal surface. Mucoadhesion, defined here are the specific adhesive force arising from interpenetration and attraction between formulation polymers and the mucins, is multifaceted, and therefore more difficult to predict and measure (60, 61).

In this study, a systematic approach was used to isolate the contribution of these formulation–mucin interactions to the total adhesion. A mucin-gelatin substrate was used as an analog for the mucin rich gel-layer of nasal mucus (17). The presence of mucins lowered the contact angle for all formulations tested, increasing wettability and reducing the time to flow down an inclined surface. However, a time dependent relationship was observed for gellan containing formulations on mucin functionalized surfaces, seen more clearly when normalized to gelatin only substrates. This was probed initially through contact angle measurements, however there was no significant time dependence for any gellan-containing formulation on the mucin gelatin surface. This showed that the rearrangement of the polymer to lower the interfacial tension was not responsible for retarding the flow speed, and it is therefore suggested that increased adhesion was due to polymer–mucin interactions developing over time. Small deformation rheology was used to probe this hypothesis, again normalizing against data from a gelatin only substrate to remove all non-mucospecific interactions. The increase in relative complex viscosity with time is therefore believed to be due to increased adhesion at the spray-substrate interface, where polymer–mucin interactions developing over time increase resistance to flow (62). The relative increase in the complex viscosity of gellan was more than three-fold that of carrageenan, suggesting that it is significantly more mucoadhesive. A key reason for this may be that gellan has far more hydroxyl groups than iota carrageenan (Figure 2), which gives a greater capacity for hydrogen bonding with the mucins (63, 64), though other secondary bonds and steric interactions may also play a role. The retention of this mucoadhesive effect in the mixed system shows that gellan is a good excipient to improve the mucoadhesion of carrageenan, potentially allowing a greater retention time within the nasal cavity, and thus prolonged antiviral effectiveness.

## Conclusions

Carrageenan exhibits broad, non-pharmacological antiviral properties, as well as a host of translational advantages making it an ideal candidate for novel antiviral nasal sprays. However, carrageenan solutions display poor spraying and low mucoadhesion, reducing their usefulness for this application. As such, incorporation of gellan as an excipient to enhance these properties was investigated. It was found that viscoelastic relaxation time was the key predictor of spray coverage in these systems, while viscosity and surface tension, which have been previously reported to drive spray formation, were of minor importance. Gellan gave better sprayability than carrageenan, and a mixture of the two polymers gave greater coverage than either single polymer formulation, possibly owing to the formation of a phase separated blend reducing the relaxation time of the system. Gellan also exhibited greater adhesion to mucin containing substrates than carrageenan, and the significantly higher time dependence of this interaction suggests specific interactions between the gellan and mucins, a property extended to mixtures of the two polymers. This data therefore suggests that gellan has great potential as an excipient to improve both sprayability and mucoadhesion in antiviral carrageenan nasal spray formulations.

## Data Availability Statement

The raw data supporting the conclusions of this article will be made available by the authors, without undue reservation.

## Author Contributions

All authors listed have made a substantial, direct and intellectual contribution to the work, and approved it for publication.

## Conflict of Interest

The authors declare that the research was conducted in the absence of any commercial or financial relationships that could be construed as a potential conflict of interest. RM and LG hold a patent filing that describes the formulation of a gellan/carrageenan spray to combat viral infection.
